# Quantifying perfusion-related energy losses during magnetic resonance-guided focused ultrasound

**DOI:** 10.1186/2050-5736-3-S1-O103

**Published:** 2015-06-30

**Authors:** Christopher Dillon, Robert Roemer, Allison Payne

**Affiliations:** 1 University of Utah, Salt Lake City, Utah, United States

## Background/introduction

The focused ultrasound power required for successful ablation of uterine fibroid tissue varies substantially between patients and within single treatments.[1,2] Fibroids with high signal intensity in pretreatment T2-weighted MR images have been shown to require increased power to achieve adequate temperature elevation for ablation;[2,4] thus, T2-weighted signal intensity has been suggested as a predictor of MRgFUS treatment response.[2,3] Physiologically, the high intensity of T2-weighted MR images of uterine fibroids may represent vascularization, fluid-rich tissues, or degeneration.[4,6] By quantifying perfusion-related energy losses (Qb) during MRgFUS treatments, this study is the first step in linking perfusion-related energy losses with MR perfusion imaging. This knowledge could be used to improve biothermal modeling of MRgFUS fibroid treatments and as a potential independent predictor of treatment response and outcome.

## Methods

**Figure 1:**
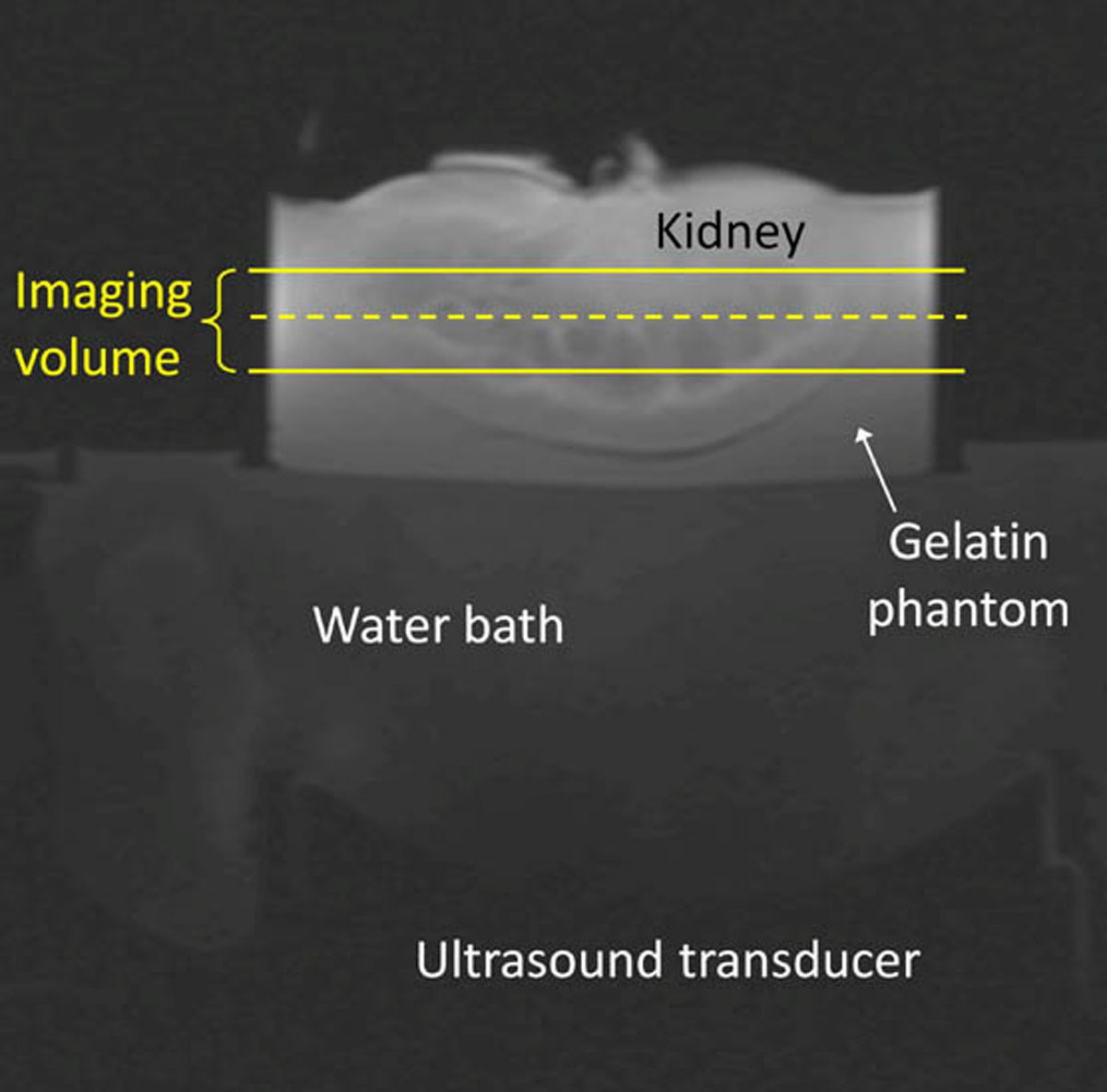
Experimental setup for MRgFUS heating of *ex vivo* perfused porcine kidney embedded in a gelatin phantom. Solid lines indicate the 3D MR temperature imaging volume and the dashed line indicates the location of the coronal magnitude image seen in figure 2.

**Figure 2:**
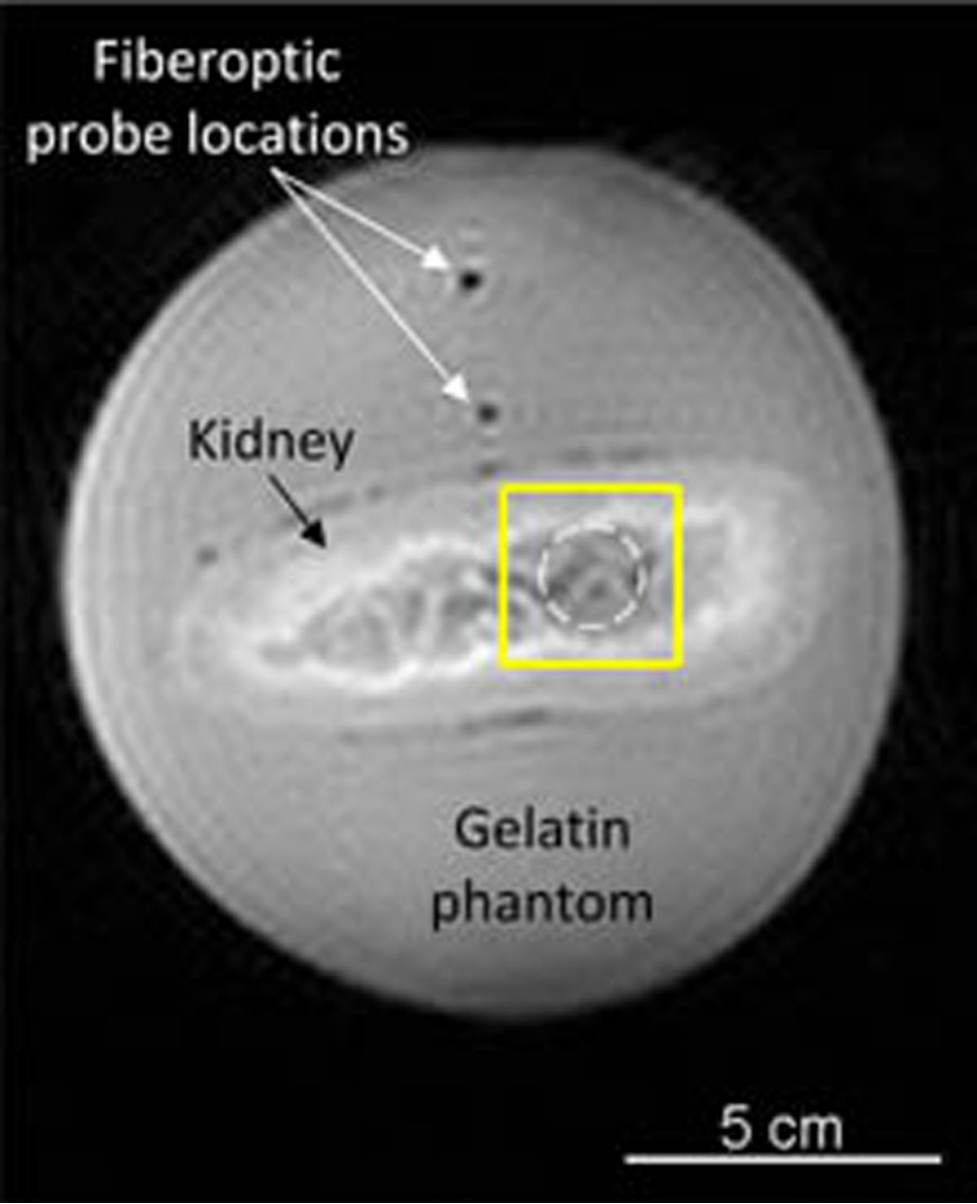
Coronal magnitude image obtained during MRgFUS heating. Fiberoptic probes measured the background temperature. The dashed line indicates the circular heating region and the solid line identifies the region of interest for data presented in figures 3 and 4.

**Figure 3:**
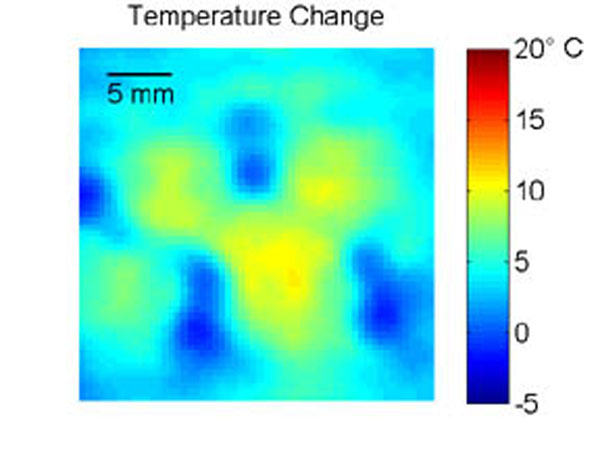
Temperature change resulting from 120 s FUS heating and 30 s cooling with a flow rate of 40 mL/min. Areas of increased cooling (blue) are likely locations of discrete vasculature.

Experiments were performed in *ex vivo* porcine kidneys perfused with a heparin- H_2_O solution in variable flow (0, 20, 40 mL/min) situations and embedded in a gelatin phantom (Figure 1). Heating was achieved by electronically steering a phased-array ultrasound transducer (256 elements, f=1 MHz) in an 8 mm-radius circle for 120 s (Figure 2). MR temperature data (Figure 3) were acquired with a 3T Siemens Trio MRI (3D segmented-EPI, TR/TE=30/11 ms, FA=15°, EPI factor=9, 2x2x3 mm3, 3.3 s acquisition, ZFI to 0.5-mm isotropic spacing). Based on conservation of energy principles, deviation of a thermal model that excludes perfusion effects from the experimental temperatures was used to quantify Qb. Estimates of Qb were obtained at the time of each MR acquisition during cooling, transformed into perfusion values via the Pennes bioheat transfer equation,[7] and averaged to mitigate the effects of noise.

## Results and conclusions

**Figure 4:**
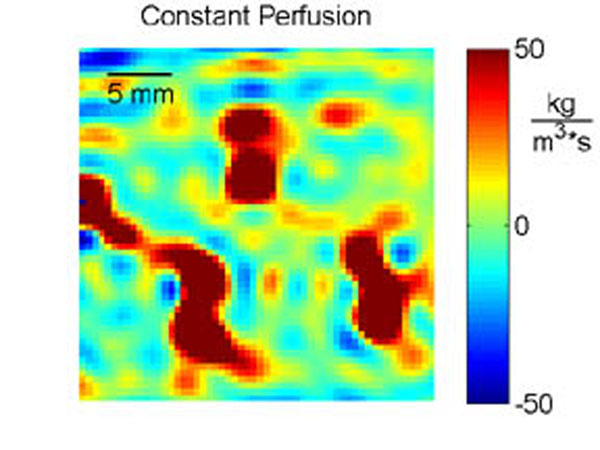
Perfusion values averaged for the first 30 s of cooling. High perfusion values (red) correspond to regions of increased cooling (blue in figure 3) and likely indicate locations of discrete vasculature.

High perfusion values (Figure 4) correspond to regions of increased cooling (Figure 3) and likely indicate locations of discrete vasculature. Constant, uniform perfusion values ranged from -0.7–0.1, 1.6–3.9, and 3.4–4.4 kg/m3/s for 0, 20, and 40 mL/min flow rates, respectively, following anticipated trends with perfusion approximately zero for the no flow case and increasing with flow rate. Future work will relate MR perfusion imaging to Qb, which should eliminate the need for tissue heating for improved biothermal modeling. This study demonstrates that obtaining perfusion estimates from 3D MR temperature data during MRgFUS is feasible and has the potential to improve biothermal models of MRgFUS fibroid treatments.

## References

[B1] McDannold N Uterine Leiomymomas: MR Imaging-based Thermometry and Thermal Dosimetry during Focused Ultrasound Thermal Ablation Radiology 2006 240 1 263 272 10.1148/radiol.2401050717 16793983 PMC1850234

[B2] Lenard Z Uterine Leiomyomas: MR Imaging-guided Focused Ultrasound Surgery-Imaging Predictors of Success Radiology 2008 249 1 187 194 10.1148/radiol.2491071600 18695211 PMC2657858

[B3] Funaki Magnetic resonance-guided focused ultrasound surgery for uterine firboids: relationship between the therapeutic effects and signal intensity of preexcisting T2-weighted magnetic resonance images Am J Obstet Gynecol 2007 196 2 e1 6 10.1016/j.ajog.2007.01.029 17306674

[B4] Fennessy F Tempany C An Update on Magnetic Resonance Guided Focused Ultrasound Surgery (MRgFUS) of Uterine Fibroids Curr Radiol Rep 2013 1 2

[B5] Swe Radiat Med 1992 1287735

[B6] Oguchi O Prediction of histopathologic features and proliferative activity of uterine leiomyoma by magnetic resonance imaging prior to GnRH analogue therapy: correlation between T2-weighted images and effect of GnRH analogue J Obstet Gynaecol 1995 21 2 107 17 10.1111/j.1447-0756.1995.tb01083.x 8556572

[B7] Pennes H Analysis of tissue and arterial blood temperature in the resting human forearm J Appl Physiol 1978 1 93 122 18887578 10.1152/jappl.1948.1.2.93

